# Multiservice-Based Traffic Scheduling for 5G Access Traffic Steering, Switching and Splitting

**DOI:** 10.3390/s22093285

**Published:** 2022-04-25

**Authors:** Xinran Ba, Libiao Jin, Zengrui Li, Jianhe Du, Sidong Li

**Affiliations:** 1 State Key Laboratory of Media Convergence and Communication, School of Information and Communication Engineering, Communication University of China, Beijing 100024, China; baxinran@cuc.edu.cn; 2 School of Information and Communication Engineering, Communication University of China, Beijing 100024, China; zrli@cuc.edu.cn (Z.L.); dujianhe1@gmail.com (J.D.); 3 Datang Mobile Communication Equipment Co., Ltd., Beijing 100083, China; lisidong@catt.cn

**Keywords:** ATSSS, multipath transport, multiservice, effective network capacity

## Abstract

As a key enabler of the access traffic steering, switching and splitting (ATSSS) feature, multipath transport can leverage the simultaneous use of several network paths and support seamless failover to improve both communication throughput and resilience. Therefore, a traffic scheduling strategy is necessary to determine the best network path combination that may improve the performance of multipath transport. To address this need, we developed a multiservice-type based transmission (MSTT) traffic scheduling optimization strategy, which involves three steps. First, the user equipment (UE) selects the number of data stream transmission paths, considering the service utility function, and either transmits all data streams via the 3GPP network or sends two streams, one via the 3GPP network and the other via the non-3GPP network. Second, the proposed method is used to select the transmission path for each data stream based on load balancing. Finally, an algorithm for optimizing traffic scheduling is formulated by applying the convex optimization problem to maximize the effective network capacity under a Delay Quality of Service (DQoS) constraint. The proposed traffic scheduling strategy is validated through simulation experiments. The results indicate that user satisfaction and effective capacity realized are always better than when using the always-best-connected and fixed-ratio power-allocation algorithms.

## 1. Introduction

To meet diverse user needs for information communication, satisfy the associated bandwidth requirements, and fully utilize the characteristics and advantages of various types of access networks, such as long-term evolution (LTE), new radio (NR), and wireless local-area network (WLAN), heterogeneous network convergence has become popular in fifth-generation (5G) networks [1]. The Access traffic steering, switching, and splitting (ATSSS) feature offers significant advantages for existing mobile networks, and is the principal solution for achieving multi-network convergence. ATSSS is an optional feature that may be supported by user equipment (UE) and 5G Core networks. ATSSS also enables a multi-access protocol data unit (PDU) connectivity service, which can exchange PDUs between the UE and a data network by simultaneously using one third-generation partnership project (3GPP) access network and one non-3GPP access network [2]. Among non-3GPP access networks, WLAN has become the preferred method for convergence with cellular networks in the communications industry owing to advantageous features, such as its high bandwidth, low cost, and flexible deployment [3,4,5,6]. Therefore, technology for the NR and WLAN has attracted significant attention from industries and academia due to its potential to alleviate data traffic congestion in licensed frequency bands using unlicensed frequency bands [3]. The two types of networks (3GPP and non-3GPP) exhibit strong complementarity, using unlicensed frequency bands to compensate for insufficient cellular network bandwidth and relying on the high-security features of cellular networks to complete user-identity-access authentication. The authors of [4] have proposed that data offloading should be seriously considered in 4G LTE networks. To date, several studies on LTE and WLAN aggregation (LWA) have been conducted to explore different interface designs and mechanisms for transport, resource-scheduling, and security [7,8,9,10,11,12]. Additionally, NR and WLAN aggregation (NWA) technology has been developed, which enables UE to implement data offloading and aggregation on both WLAN and NR network links within a 5G-specific scenario. However, its technical feasibility and network capacity gain has not yet been explicitly verified.

For the design, demonstration, and verification of an NWA system, the data splitting and merging mechanism would be the crucial step. During this process, we must consider the performance characteristics of the underlying link between the NR and WLAN, and efficiently utilize the capabilities of the physical layers of multiple networks by reasonably balancing load and allocating power and rate. It is also necessary to reduce delay in data convergence packet aggregation protocol to improve user experience.

### 1.1. Background

**Figure 1:**
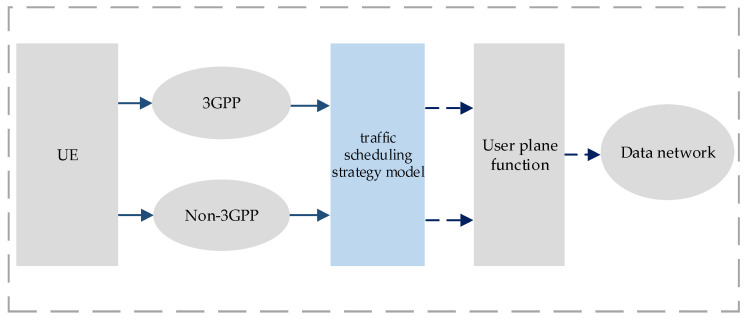
Architecture for ATSSS support.

With the introduction of the latest 3GPP R17, 5G system architecture was expanded to support ATSSS, as shown in Figure 1. According to the ATSSS rules, provided by the traffic scheduling strategy model, the UE supports traffic steering, switching, and splitting across 3GPP and non-3GPP access. Furthermore, several theoretical studies using NWA have been implemented. The data transmitted from the UE to the data network have parallel links to the 3GPP (NR) and non-3GPP (WLAN) networks. Therefore, following a reasonable traffic scheduling strategy, the UE can choose between passing all its data in a single stream through the NR network or dividing it into two streams, one that passes through the NR network and another that passes through the WLAN. Given that NR networks and WLANs exhibit significantly different network coverage, signal-to-noise-ratio (SNR) reliability, and user distance, a reasonable allocation of the power and network resources is necessary to optimize the network capacity [13].

The service type of the UE determines the characteristics of its uplink data. Additionally, the development of new mobile applications gives rise to different services, including traditional voice services and multimedia services, such as data and video streaming. Thus, user service requirements are gradually demanding more broadband, diversification, and individualization. Moreover, different Quality of Service (QoS), i.e., data transmission rates and time delays, are required for different technologies and terminals [14,15,16]. Access to a technology that meets all user-service requirements is difficult, and the contradiction between complex service requirements and inefficient resource scheduling has become increasingly prominent. Conversely, services in heterogeneous converged networks face burstiness, which results in an asymmetric network service distribution. Presently, users mostly connect to a single network to transmit all business data according to their personal preferences and habits. This can easily lead to problems, such as access congestion and low QoS, in a single network, while large amounts of alternative network resources remain idle.

### 1.2. Related Work

Multipath management, traffic scheduling, and congestion control are the main topics being studied by the 3GPP SA2 working group. There are also technical difficulties in implementing the ATSSS feature currently under research. Fortunately, traffic scheduling methods in LWA and multipath transmission control protocol (MPTCP) have received significant research attention over the years. Owing to the similarity between LWA and ATSSS, traffic scheduling strategies for the former can be used as a reference for the development of new ones for the latter.

In [17], a novel radio access technology (RAT) selection method was proposed that can maximize the total throughput by simultaneously selecting optimal RATs for a group of UEs. In [18], it was proved essential that the network should intelligently switch a data radio bearer (DRB) to utilize either LTE or Wi-Fi. The authors of [18] proposed two types of switched LWA policies: guaranteed bandwidth and equal sharing. Their study indicated that switched LWA can effectively reduce the blocking probability of the DRBs. Both [17,18] provided efficient and high-capacity mode selection methods and solutions to the handover problem between different access technologies that do not support the simultaneous operation of multiple access technologies. The traffic load balancing and resource-allocation scheme proposed in [19] is set to play a crucial role in leveraging the dense and increasingly heterogeneous deployment of multi-radio wireless networks. A scenario is considered in [19], where the traffic for each user may be split across macro-cells or small Wi-Fi cells, connected by non-ideal backhaul links. The fraction of the user’s traffic sent over macro-cells is proportional to the ratio between the peak capacity of that macro-cell and the throughput of the small cell. Reference [20] focuses on the MPTCP scheduler, with the goal of providing a good user experience for latency-sensitive applications when interface quality is asymmetric. Two novel scheduling techniques are presented in [20], which reduce web object transmission times and provide faster communication for interactive applications, compared to MPTCP’s default scheduler. However, Refs. [19,20] only focus on the performance with one user, and neither considers fairness between all users, nor achieves an optimal system. In a previous study [21], linear programming techniques were used to optimize the bandwidth of a licensed spectrum, using the bandwidth of an unlicensed spectrum to maximize the overall network throughput. In study [22], the convex optimization theory was applied to the power of a licensed spectrum, and the duration of an unlicensed one was used to maximize the total utility for users.

In [23], delay-tolerance service was proposed for the first time for LWA, and a cross-system learning method was also proposed to optimize power, cell-range extension bias, sub-band selection, and service scheduling. In study [24], a semi-Markov model, based on a distributed coordinated function channel-access mechanism, was proposed to establish a Wi-Fi network between closely related network resources capable of meeting the QoS requirements. Musavian et al. [25] proposed a rate-efficient power allocation strategy for delay outage limited applications with constraints on energy-per-bit consumption of the system. Roy et al. [26] proposed computationally efficient algorithms based on threshold structures for the association and offloading of users in LWA HetNet.

Although traditional radio resources include user-transmit power, bandwidth, time, and spectrum, since the development of 5G, the focus of traffic scheduling has shifted to resource blocks and transmit power. In the traditional traffic scheduling algorithm, the optimal allocation of network resources is mostly achieved by maximizing the total network throughput. However, for a multiservice network, when a user requests distinct services simultaneously, the delay-type QoS (DQoS) characteristics that match the service requirements must also be considered when scheduling traffic. Recently, the application of an optimal resource-allocation algorithm, based on the maximization of the effective network capacity, yielded good performance in several networks. The use of Karush–Kuhn–Tucker (KKT) conditions to maximize the effective network capacity for mobile video traffic has been proposed [27]. The use of a semi-Markov model has been proposed to derive the effective network capacity under LWA, which is achieved using the delay constraint to offload the network traffic from a licensed frequency band to an unlicensed one [28]. These algorithms demonstrated the feasibility of fusing licensed spectrum-based 3GPP networks with unlicensed spectrum-based non-3GPP networks to realize superior network capacity. With the development of ATSSS technology and the rise of multiservice user applications, major telecommunications operators have invested considerable amounts of resources in the development and verification of NWA technologies, including the scheduling and optimization of resources.

Spurred by both economic and operational considerations, and by environmental concerns, energy efficiency has now become a key pillar in the design of communication networks [29]. In [30], a deep reinforcement learning-based power control scheme is proposed for improving the system-level energy EE of two-tier 5G heterogeneous and multi-channel cells. The algorithm aims to maximize the EE of the system by regulating the transmission power of the downlink channels and reconfiguring the user association scheme. By introducing a machine learning (ML) algorithm from classical RL to solve the objective problem, the authors of [31] propose a joint power control and channel allocation scheme, based on combining an RL algorithm with statistical CSI, to reduce interference adaptively. In the future, we will incorporate ML and RL methods into our research by training agents to “learn” favorable policies to increase the effective capacity of the system, and thus improve EE.

In our previous study [32], we optimized network throughput and various business utility functions to obtain optimal power- and bandwidth-allocation mechanisms. Experimental simulations also confirmed that the use of NWA could effectively improve network throughput and user satisfaction. However, the parameters of the wireless channel are constantly changing with time, frequency, and space making it difficult to deterministically guarantee service quality in an actual wireless communication system. With the continuous development of statistical service quality assurance theories, the resolution of deterministic service quality assurance in traditional wireless networks is improving. On this basis, this study was focused on meeting the various business QoS requirements of users. In wireless communication, end-to-end delay is a parameter that directly affects QoS and user experience. Therefore, delay cannot be ignored in wireless communication channels, and the guarantee of end-to-end delay has become a crucial objective of heterogeneous multi-connectivity networks. Although traditional channel modeling, with the primary objective of maximizing network throughput, does not reflect the delay index, the effective network capacity derived from the effective network bandwidth in a wired network describes the relationships among service delay, bandwidth, power, and data rate.

### 1.3. Our Contribution

In this study, we considered and investigated an optimization problem related to traffic scheduling in NWA systems. The novelty of this study is highlighted by the following facts: (1) Our algorithm breaks the existing strategy, which offers users the possibility to connect to only one type of network (3GPP or non-3GPP) so that the transmitted data can be dynamically transmitted over one or two networks simultaneously, based on the service type; (2) The data stream transmission corresponding to each service can avoid high-load paths and choose alternative low-load paths; (3) Through distributed power- and bandwidth-allocation strategies, the effective capacity of the system can be maximized, while satisfying the specific service delay requirements of different users.

Additionally, this study makes the following contributions:

1. A smart mode selection module was employed to choose between 3GPP and non-3GPP access. A model for the selection of the transmission path, based on the utility function, was developed.
2. A method for determination of the suitable service network, based on the network load, which quickly and effectively discards high-load networks, was developed. This method comprises three steps: (a) determination of the service network candidate set; (b) elimination of the poor link quality network; and (c) elimination of the high-load network, based on a sigmoid function.
3. An effective network capacity maximization problem was formulated under specific DQoS, statistical bandwidth and power constraints. Using the Lagrangian function and the sub-gradient algorithm, the original problem was solved and an optimal resource allocation solution for 3GPP access and non-3GPP was derived.

The rest of this paper is organized as follows. Section 2 introduces the system model, including the network and service utility components, as well as the calculation of the effective network capacity. In Section 3, we describe the proposed resource optimization scheme, which is divided into three steps: (1) determination of the number of connection links; (2) selection of the transmission path; and (3) distribution of resource allocation. In Section 4, we show the convergence of the link adaptations and provide simulation results corresponding to our scheme. Finally, Section 5 presents the conclusions drawn from the simulation results.

## 2. System Model

A multi-cell network with a set of UEs, $M=\left\{m_{1},\;m_{2}\cdots m_{m}\right\}$, which need to send their data to the core network, was considered. The set of all the NR base stations (BSs) was denoted by $L=\left\{l_{1},\;l_{2},\cdots l_{l}\right\}$, and the set of all the WLAN access points (APs) was denoted by $W=\left\{w_{1},\;w_{2},\cdots w_{w}\right\}$. Each UE could accommodate two parallel links, one to the NR network and another to the WLAN. Additionally, since WLAN cannot exist independently (without association with the NR network) in our model, a UE maintains its connection to the NR network through its first link, which is referred to as the dedicated link, and ensures the transmission of its control commands. The NR network chosen by each UE as its dedicated link is then introduced in the next module. On its second link, referred to as the adaptive link, the UE may choose the WLAN AP to send data to the core network. The WLAN chosen by each UE as its adaptive link is the one from which the strongest pilot signal is received on a downlink control channel. All the links in the network (i.e., UE to NR and UE to WLAN) can allocate their transmit power and, consequently, their data rate. Considering the available power vector of the UEs as $P_{\max}=[P_{\max,1},\cdots P_{\max,m}]$ (unit: W), the transmit powers of the UEs, with respect to their dedicated and adaptive links, are represented by the vectors $P_{l}=\left[P_{1}^{\left(l\right)},P_{2}^{\left(l\right)}\cdots P_{m}^{\left(l\right)}\right]$ and $P_{w}=\left[P_{1}^{\left(w\right)},P_{2}^{\left(w\right)}\cdots P_{m}^{\left(w\right)}\right]$, respectively, where $P_{i}^{\left(l\right)}+P_{i}^{\left(w\right)}\leq P_{\max,i}$.

### 2.1. Network Model

The channel gain between UE, i, and the intended receiver, which depends on several factors, such as shadowing, path loss, and fading, is represented as $h_{i,l}$($h_{i,w}$). We ignored the band allocation differences between cell-edge users and central users. The WLAN adopted the carrier sense multiple access/collision avoidance (CSMA/CA) mechanism, and the main purpose of converging the WLAN was to reduce the burden on the NR network. To better manage intra-cell interference, we assumed that the NR dedicated links may suffer intra-cell interference due to frequency reuse, but WLAN links operate on channels that are not on the same frequency as NR. Therefore, only limited noise is present. For UE, i, with $x_{i}^{\left(l\right)}$ and $x_{i}^{\left(w\right)}$ as the unit-power complex-valued input symbols sent over the dedicated and adaptive links, respectively, the output symbols $y_{i}^{\left(l\right)}$ and $y_{i}^{\left(w\right)}$ can be expressed as follows [33]:

(1) $$y_{i}^{\left(l\right)}=h_{i,l}\sqrt{P_{i}^{\left(l\right)}}x_{i}^{\left(l\right)}+\sum_{\forall j\neq i}h_{j,l}\sqrt{P_{j}^{\left(l\right)}}x_{j}^{\left(l\right)}+v_{i}^{\left(l\right)},\;y_{i}^{\left(w\right)}=h_{i,w}\sqrt{P_{i}^{\left(w\right)}}x_{i}^{\left(w\right)}+v_{i}^{\left(w\right)},$$

where $v_{i}^{\left(l\right)}$ and $v_{i}^{\left(w\right)}$ denote the zero-mean complex Gaussian noise on the dedicated (NR) and adaptive (WALN) links, respectively. Owing to the existing frequency characteristics, there was no interference between the dedicated and adaptive links. Thus, we assumed that interference exists only when users access the same bandwidth resource block, and the effective interference is then defined as follows:

(2) $$N_{i}^{\left(l\right)}=\sum_{j=1}^{m}h_{j,l}P_{j}^{\left(l\right)}+n_{s},\;N_{i}^{\left(w\right)}=n_{w},$$

where $n_{s}=Var[v_{i}^{\left(l\right)}]$ and $n_{w}=Var[v_{i}^{\left(w\right)}]$ are the thermal noise powers. The corresponding signal to interference plus noise ratio can be expressed as follows:

(3) $$\gamma_{i}^{\left(l\right)}=\frac{P_{i}^{\left(l\right)}}{N_{i}^{\left(l\right)}}=\frac{P_{i}^{\left(l\right)}}{\sum_{j=1}^{m}h_{j,l}P_{j}^{\left(l\right)}+n_{s}},\;\gamma_{i}^{\left(w\right)}=\frac{P_{i}^{\left(w\right)}}{N_{i}^{\left(w\right)}}=\frac{P_{i}^{\left(w\right)}}{n_{w}}.$$

### 2.2. Service Utility Model

The utility function is derived from economics and is used to express the quantitative relationship between the utility obtained by consumers during consumption and the combination of commodities consumed, i.e., it measures the degree of satisfaction that consumers obtain from the consumption of a given combination of commodities. In recent years, utility functions have been increasingly adopted in research on wireless network resource allocation to effectively characterize the users’ preferences and network performances [34,35]. The utility function is also used in delay-critical businesses. For example, Ref. [36] considered a multi-path routing problem of maximizing the aggregate user utility over a multi-hop network. Furthermore, Ref. [37] studied delay-optimal packet scheduling strategies for a M2M uplink, with heterogeneous data arriving at a M2M Application Server via multiple M2M Aggregators. In these studies, the utility function measures the impact of any time delay on user satisfaction. In our research, the utility function is used for performance evaluation of the end-to-end communication delay. Generally, UEs initiate multiple services simultaneously, and the completion of each service is subject to different QoS requirements. Since the utility function can quantify QoS and measure user satisfaction in recent years it has increasingly been employed to realize resource allocation, power control, and flow control [38,39]. We define three utility functions, namely the constant bit rate (CBR), download service (DS), and variable bit rate (VBR). $U_{i}$ and $V_{i}$ represent the utility function and transmission rate, respectively, corresponding to service i, and $Td_{i}$ represents the threshold that service i must reach to be successfully completed.

CBR

Usually, CBR services do not require a high rate and only need to meet a threshold to ensure normal data transmission. However, if this threshold is not reached, the user experience is drastically impaired. For voice services, users are more sensitive to delay than to transmission rate. Therefore, normal voice communication only needs to meet an 8 Kbps threshold; however, if this threshold is not reached, the communication is interrupted, and this further impacts the user experience. The utility function for CBR services is represented by the following step function:

(4) $$U_{cbr}=\left\{ \begin{array}{l} 0\begin{array}{ccc} , & & ifV_{cbr}<Td_{cbr}, \end{array}\\ 1\begin{array}{ccc} , & & ifV_{cbr}\geq Td_{cbr} \end{array}. \end{array}\right.$$

DS

DSs, including file transfer and multimedia streaming, have relatively high throughput requirements. As the data rate increases, user satisfaction increases and user sensitivity reduces. Therefore, the relationship between data rate and user satisfaction is not linear. Thus, the utility function of data services is expressed as follows:

(5) $$U_{ds}=1-e^{-\frac{V_{ds}}{Td_{ds}}}.$$

VBR

Although the usefulness of VBR services improves with increase in data rate, much like with DSs, VBR services have more stringent requirements for packet loss rate. For example, the utility of video services changes smoothly when the data rate is very low or very high. This is because at very low data rates packet loss rate is high and increases the link failure rate, while at very high data rates packet loss rate is low and can ensure video quality. We use a sigmoid function to express the utility function of VBR services as follows:

(6) $$U_{vbr}=\frac{1}{1+e^{-\omega \left(V_{vbr}-Td_{vbr}\right)}}.$$

### 2.3. Effective Network Capacity

**Figure 2:**
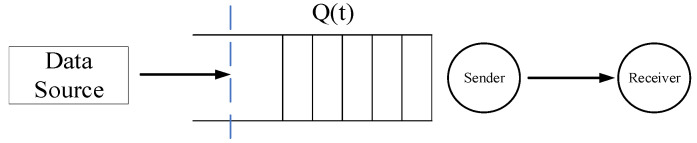
Queuing system model.

Most services use data packet-switching technology, which requires a guaranteed QoS to ensure the order of control-information resources during data transmission across the network. An effective and practical QoS support mechanism requires an accurate and simple channel model [40]. For this reason, it is necessary to model the wireless channel based on QoS indicators, such as data rate, delay, and delay violation probability. The queuing system model in Figure 2 shows that the source traffic and the network service are matched using a first-in first-out buffer (queue). Thus, the queue prevents the loss of packets that could occur when the source rate exceeds the service rate, at the expense of an increased delay. Using the effective capacity and statistical delay service quality parameters proposed in [41], the end-to-end delay of each user is modeled. The model aims to characterize wireless channels in terms of functions that can be easily mapped to link-level QoS metrics, such as delay-bound violation probability. This transmission model is also the theoretical basis of our heterogeneous network multiservice transmission. During data-packet queuing, multiple services, such as those pertaining to voice, data, and video, are ordered, and different network resources are allocated to each.

Consequently, we assume that end-to-end communications are modeled based on a queuing system, wherein the DQoS index, θ, is given by Equation (7) [42]:where

(7) $$-\underset{x\to \infty }{\lim}\frac{\log\left(\mathrm{Pr}\left\{Q\left(\infty \right)>x\right\}\right)}{x}=\theta ,$$

x represents the queue-length threshold. According to the large-deviation theory, the probability that the queue-length process Q(t) exceeds the threshold x decreases exponentially as x increases. Additionally, the DQoS index, θ, is used to measure the exponential decay rate of the current link-violation service; the larger the θ value, the greater the possibility that the link satisfies strict delay service requirements. Conversely, the smaller the θ value, the greater the possibility that the channel is guaranteed only by a loose delay service [43]. Furthermore, Equation (7) shows that the probability that the queue-length violates the threshold, x, can be expressed as follows [44]:

(8) $$\mathrm{Pr}\left\{Q\left(\infty \right)>x\right\}=e^{-\theta x}.$$

The instantaneous data rate on link i, $R_{i}$, which can be determined using Shannon’s formula, is expressed as follows:

(9) $$R_{i}=B\log_{2}\left(1+P_{i}\gamma_{i}\right).$$

where B represents the bandwidth of link i, $P_{i}$ represents the transmission power allocated by the user to link i, and $\gamma_{i}$ represents the instantaneous SNR obtained by the uplink i. Assuming that $\theta_{i}$ represents the DQoS index for link i, the effective network capacity $E\left(\theta_{i}\right)$ of link i can be represented according to Equation (10), where $E_{\gamma_{i}}\left(\cdot \right)$ represents the expectation of $\gamma_{i}$.

(10) $$E\left(\theta_{i}\right)=-\frac{1}{\theta_{i}}\log\left(E_{\gamma_{i}}\left\{e^{-\theta_{i}R_{i}}\right\}\right)=-\frac{1}{\theta_{i}}\log\left(E_{\gamma_{i}}\left\{e^{-\theta_{i}B\log_{2}\left(1+P_{i}\gamma_{i}\right)}\right\}\right).$$

## 3. Multiservice Type-Based Transmission (MSTT)

**Figure 3:**
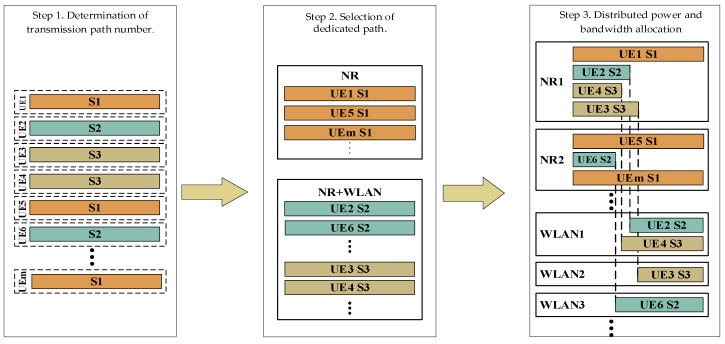
Multiservice traffic scheduling model.

Based on the foregoing DQoS guarantee mechanism, we proposed a scheme to optimize resource allocation by maximizing the total effective network capacity of the NR and WLAN links. The algorithm comprises three steps. First, the UE determines the number of data transmission paths to either (a) transmit its two data streams through the NR or (b) send one stream through the NR and the other through the WLAN. Then, a method to select the transmission path based on load balancing is used to plan the data streams transmission path of each service. Finally, a traffic scheduling scheme under the delay service quality constraint is developed to determine user power decomposition and bandwidth allocation. A schematic diagram of the algorithm is shown in Figure 3.

The steps are described as follows:

Step 1. Determination of transmission path number.

The total utility function of all services is expressed as follows:

(11) $$U_{all}=\sum_{r\in R}\log_{2}\left(U_{r}\right),$$

where r∈R(CBR,DS,VBR). To maximally satisfy the user QoS requirements and obtain the maximum overall utility, in the MSTT scheme, it is necessary to first ensure that the rate of the CBR service exceeds the basic transmission threshold. Therefore, according to the characteristics of the CBR utility function, all the CBRs are set to adopt a single connection. Additionally, users implement a single connection when the WLAN network is occupied. Thus, the number of upload paths for all the service data streams is expressed as

(12) $$\left\{ \begin{array}{l} 1\begin{array}{ccc} & & CBRorWLANcollision \end{array}\\ 2\begin{array}{ccc} & & \left\{DS,VBR\right\} \end{array}andWLANidle \end{array}\right.$$

Step 2. Selection of dedicated path.

In this subsection, a dynamic dedicated path selection scheme, which allows a user to dynamically adjust the dedicated path based on channel-state information and NR BS load, is proposed. The UE estimates the channel quality of the serving BS and the neighboring cells using the reference signal received power (RSRP) report, which is the most common parameter in mobility decisions for heterogeneous networks. The averaged UE measurement, $M_{u,c}$, of UE u from BS c at the $n^{th}$ time step is calculated as

(13) $$M_{u,c}=\left(1-a\right)\cdot M_{u,c}\left(n-1\right)+a\cdot R_{u,c}\left(n\right),$$

where $R_{u,c}\left(n\right)$ represents the RSRP instantaneously measured by UE u from BS c, and a is the filter coefficient configured by the network [45,46]. It should be clarified that the $M_{u,c}$ of each UE is acquired only once, whereas the location of the UE is updated at every time step.

The UE u first initializes the uplink candidate set according to a preset threshold, then removes the BSs with poor channel quality based on the relative threshold, and finally removes the overloaded BSs. This not only ensures the throughput requirements of UE u but also achieves load balancing for the entire system.

(1) Uplink candidate set initialization: For each u, we assumed that, if $M_{u,c}$ is greater than the threshold $M^{th}$, the BS c can be added to the candidate set of UE u, i.e., $\underset{c\in A_{u}}{\min}\left(M_{u,c}\right)\geq M^{th}$. Conversely, if $M_{u,c}$ is less than $M^{th}$, no connection between UE u and BS c can be established. Considering the typical urban scene layout and the sensitivity of the UE receiver, $M^{th}$ is usually set to −109 dBm.

(2) Removing weak BSs: Very weak links may not yield additional benefits but unnecessarily increase complexity. If the difference between $M_{u,c}$ and the strongest RSRP BS is greater than the removal offset, $M^{rmv}$, BS c will be deleted from the candidate set. However, if it can remain in the candidate set, the following condition must be satisfied:

(14) $$M_{u,c}\geq \underset{c\in A_{u}}{\max}\left(M_{u,c}\right)-M^{rmv},$$

where $A_{u}$ represents the candidate set of UE u. A previous study showed that when $M^{rmv}=9\mathrm{dB}$, the radio link failure (RLF) is fully resolved [32].

(3) System load balancing: The aforementioned threshold can effectively limit the size of the candidate set, but this limitation can be further enforced. In this study, to realize system load balancing, the following sigmoid function, which consists of a BS load, is proposed:

(15) $$S\left(L_{c},\omega ,L_{\max}\right)=\frac{1}{1+e^{-\omega \left(L_{c}-L_{\max}\right)}},$$

where ω is a parameter that affects the shape of the sigmoid function, $L_{c}$ is the load of BS c, and $L_{\max}$ represents the maximum service carrying capacity of the BS c. We assume that $L_{c}$ represents the amount of data transmitted by the current BS c.

**Figure 4:**
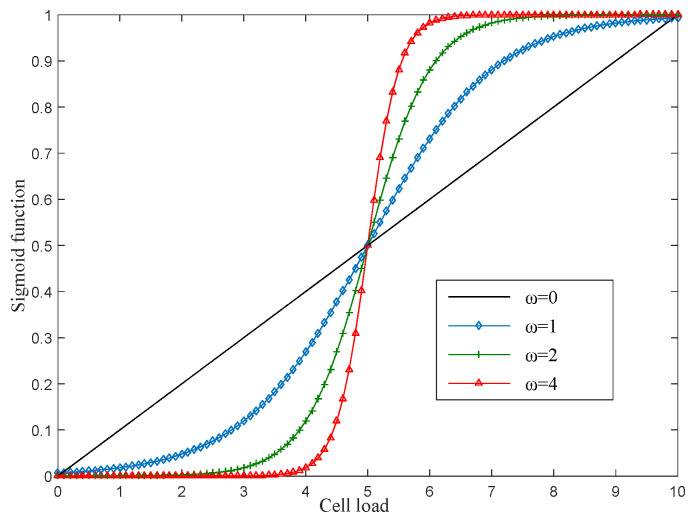
Sigmoid function family with different ω.

In Figure 4, a family of sigmoid functions with a different ω is compared with the linear increment function for $L_{\max}=5$. Cell c can remain in $A_{u}$ if it satisfies the following condition:

(16) $$\frac{M_{u,c}}{\underset{c\in A_{u}}{\max}M_{u,c}}\geq S\left(L_{c},\omega ,L_{\max}\right).$$

For BS c, Figure 4 shows that, as the BS load increases, the constraints become more stringent. In other words, the smaller the load of BS c, the greater the probability of it staying in $A_{u}$. Finally, the UE selects the link with the largest $M_{u,c}$ in the candidate set as the dedicated path. This method can effectively remove overloaded cells and achieve load balancing in the whole system.

Step 3. Distributed power and bandwidth allocation.

With the rapid growth of services required owing to the high network latency of users, improving DQoS has become an effective strategy to enhance user experience [47,48,49,50,51]. Additionally, ensuring that the delay requirements of different services on communication networks are achieved has become an urgent problem that needs to be addressed. Moreover, the combination of NR and WLAN has enabled users to achieve higher peak data-packet transmission rates. Thus, a method to maximize the total effective network capacity, while satisfying the quality requirements of different services, when the number of service paths is equal to 2 is detailed in this section.

In the model, it is assumed that there are L NR BSs occupying licensed frequency bands in the network, and that each BS bandwidth is set to $B^{L}$. Further, based on unlicensed frequency bands, it is also assumed that there are W WLAN APs in the NR BS coverage area, and each WLAN AP bandwidth is set to $B^{W}$. The channels of both frequency bands are assumed to be ideal quasistatic channels, i.e., the channel gain does not change within a given frame. The frames exhibit mutual independence; thus, users can receive ideal channel-state information. Service requests from UEs follow the file transfer protocol (FTP) data generation mechanism with a data-arrival rate α.

The proportion of data allocated to each of the two links depends on the type of service. We define the binary variable $b_{k}^{L}$ to indicate whether service k selects the NR link to upload data. If it does, $b_{k}^{L}=1$, otherwise, $b_{k}^{L}=0$. Similarly, we define the binary variable $b_{k}^{W}$ to indicate whether service k selects the WLAN link to upload data. If so, $b_{k}^{W}=1$, otherwise, $b_{k}^{W}=0$.

The effective network capacity describes the relationships among the channel and the data transmission time, power, and bandwidth under the constraints of different service DQoS conditions. It was assumed that UE i selects the two uplinks from L NR BSs and W WLAN APs via a two-step process. Thus, the effective network capacity, $E_{k}^{L}\left(\theta_{k}^{L}\right)$, for service k of UE i on the NR link is expressed as follows:

(17) $$E_{k}^{L}\left(\theta_{k}^{L}\right)=-\frac{1}{\theta_{k}^{L}}\log\left(E_{\gamma_{k}^{L}}\left\{e^{-\theta_{k}^{L}R_{k}^{L}}\right\}\right)=-\frac{1}{\theta_{k}^{L}}\log\left(E_{\gamma_{k}^{L}}\left\{e^{-\theta_{k}^{L}B_{k}^{L}\log_{2}\left(1+P_{k}^{L}\gamma_{k}^{L}\right)}\right\}\right),$$

where $\theta_{k}^{L}$ is the impact factor of the characteristics of service k on the effective network capacity of the NR link, i.e., the DQoS index of the service is $\theta_{k}^{L}$. $B_{k}^{L}$ is the uplink bandwidth allocated to service k on the NR link, $P_{k}^{L}$ is the power allocated to the UE in the NR uplink, $\gamma_{k}^{L}$ is the instantaneous rate when service k is transmitted over the NR link, and $E_{\gamma_{k}^{L}}(\cdot )$ is the expectation regarding $\gamma_{k}^{L}$.

Similarly, the effective network capacity, $E_{k}^{W}\left(\theta_{k}^{W}\right)$, for service k of UE i on the WLAN link can be expressed as follows:

(18) $$E_{k}^{W}\left(\theta_{k}^{W}\right)=-\frac{1}{\theta_{k}^{W}}\log\left(E_{\gamma_{k}^{W}}\left\{e^{-\theta_{k}^{W}R_{k}^{W}}\right\}\right)=-\frac{1}{\theta_{k}^{W}}\log\left(E_{\gamma_{k}^{W}}\left\{e^{-\theta_{k}^{W}B_{k}^{W}\log_{2}\left(1+P_{k}^{W}\gamma_{k}^{W}\right)}\right\}\right),$$

where $\theta_{k}^{W}$ is the impact factor of the characteristics of service k on the effective network capacity of the WLAN link.$B_{k}^{W}$ is the uplink bandwidth allocated to service k on the WLAN link, $P_{k}^{W}$ is the power allocated to the UE in the WLAN uplink, $\gamma_{k}^{W}$ is the instantaneous rate when service k is transmitted over the WLAN link, and $E_{\gamma_{k}^{W}}(\cdot )$ is the expectation regarding $\gamma_{k}^{W}$.

For simplicity, it was assumed that service k has the same influence on the effective network capacities of the NR and WLAN links, i.e., $\theta_{k}=\theta_{k}^{L}=\theta_{k}^{W}$. Thus, the total effective network capacity of service k on the NR and WLAN links can be expressed as follows [43]:

(19) $$E\left(\theta_{k}^{}\right)=E_{k}^{L}\left(\theta_{k}^{}\right)+E_{k}^{W}\left(\theta_{k}^{}\right)=-\frac{1}{\theta_{k}^{}}\left[\log\left(E_{\gamma_{k}^{L}}\left\{e^{-\theta_{k}^{}B_{k}^{L}\log_{2}\left(1+P_{k}^{L}\gamma_{k}^{L}\right)}\right\}\right)+\log\left(E_{\gamma_{k}^{W}}\left\{e^{-\theta_{k}^{}B_{k}^{W}\log_{2}\left(1+P_{k}^{W}\gamma_{k}^{W}\right)}\right\}\right)\right].$$

Therefore, for the multiservice NWA uplink traffic scheduling problem, the following optimization problems can be established as Equation (20):

(20) $$\underset{P^{*},B^{*}}{\arg\max}\left\{\sum_{k=1}^{k=K}-\frac{1}{\theta_{k}^{}}\left[\log\left(E_{\gamma_{k}^{L}}\left\{e^{-\theta_{k}^{}B_{k}^{L}\log_{2}\left(1+P_{k}^{L}\gamma_{k}^{L}\right)}\right\}\right)+\log\left(E_{\gamma_{k}^{W}}\left\{e^{-\theta_{k}^{}B_{k}^{W}\log_{2}\left(1+P_{k}^{W}\gamma_{k}^{W}\right)}\right\}\right)\right]\right\},$$

(21) $$s.t.\;P_{k}^{L}+P_{k}^{W}\leq P_{}^{\max},$$

(22) $$\sum_{k=1}^{k=K}B_{k}^{L}\leq B_{}^{L},$$

(23) $$\sum_{k=1}^{k=K}B_{k}^{W}\leq B_{}^{W},$$

(24) $$P_{k}^{L},P_{k}^{W},B_{k}^{L},B_{k}^{W}\geq 0.$$

The optimization objective function is a summation of multiple log functions, and, given that the constraints are linear, the problem is a convex optimization one. To reduce complexity, it is assumed that the minimum granularity of UE power allocation is represented by the link. Additionally, the constraint in Equation (21) indicates that the sum of the transmit power allocated by the user to the NR and WLAN links does not exceed the total maximum transmit power. The constraint in Equation (22) indicates that the total bandwidth on the NR link occupied by simultaneous requests for multiple services by the current user should not exceed the total uplink bandwidth of the NR. Similarly, the constraint in Equation (23) indicates that the total bandwidth on the WLAN link that is simultaneously occupied by the multiple service requests of the current user should not exceed the total uplink bandwidth of the WLAN. Moreover, the constraint in Equation (24) indicates that each service can upload data on both NR and WLAN links simultaneously. Although Equation (20) is already a convex optimization problem, it is difficult to determine the closed optimal solution using KKT conditions. Therefore, the optimization problem in Equation (20) can be transformed into a joint optimization problem of distributed optimal power and bandwidth mechanisms [43].

(1) Optimal power-allocation mechanism

If the DQoS index of service k for user i is $\theta_{k}$, the optimal power-allocation problem obtained according to Equation (20) can be expressed as follows:

(25) $$\underset{P_{k}^{L},P_{k}^{W}}{\arg\max}\left\{-\frac{1}{\theta_{k}^{}}\left[\log\left(E_{\gamma_{k}^{L}}\left\{e^{-\theta_{k}^{}B_{}^{L}\log_{2}\left(1+P_{k}^{L}\gamma_{k}^{L}\right)}\right\}\right)+\log\left(E_{\gamma_{k}^{W}}\left\{e^{-\theta_{k}^{}TB_{}^{W}\log_{2}\left(1+P_{k}^{W}\gamma_{k}^{W}\right)}\right\}\right)\right]\right\},$$

(26) $$s.t.\;P_{k}^{L}+P_{k}^{W}\leq P_{}^{\max},$$

(27) $$P_{k}^{L},P_{k}^{W}\geq 0.$$

These expressions are equivalent to the convex optimization problem, which is expressed as follows:

(28) $$\underset{P_{k}^{L},P_{k}^{W}}{\arg\min}\left\{\log\left(E_{\gamma_{k}^{L}}\left\{e^{-\theta_{k}^{}B_{}^{L}\log_{2}\left(1+P_{k}^{L}\gamma_{k}^{L}\right)}\right\}\right)+\log\left(E_{\gamma_{k}^{W}}\left\{e^{-\theta_{k}^{}B_{}^{W}\log_{2}\left(1+P_{k}^{W}\gamma_{k}^{W}\right)}\right\}\right)\right\},$$

such that Equations (26) and (27) are satisfied. Since the NR and WLAN are independent of the two links, Equation (28) becomes equivalent to the following:

(29) $$\underset{P_{k}^{L},P_{k}^{W}}{\arg\min}\left\{E_{\gamma_{k}^{L}}\left\{e^{-\theta_{k}^{}B_{}^{L}\log_{2}\left(1+P_{k}^{L}\gamma_{k}^{L}\right)}\right\}E_{\gamma_{k}^{W}}\left\{e^{-\theta_{k}^{}B_{}^{W}\log_{2}\left(1+P_{k}^{W}\gamma_{k}^{W}\right)}\right\}\right\}=\underset{P_{k}^{L},P_{k}^{W}}{\arg\min}\left\{E_{\gamma_{k}^{}}\left\{{\left(1+P_{k}^{L}\gamma_{k}^{L}\right)}^{\frac{-\theta_{k}^{}B_{}^{L}}{\ln2}}{\left(1+P_{k}^{W}\gamma_{k}^{W}\right)}^{\frac{-\theta_{k}^{}B_{}^{W}}{\ln2}}\right\}\right\},$$

where $\gamma_{k}=\left[\gamma_{k}^{L},\gamma_{k}^{W}\right]$ is the NR- and WLAN-link channel SNR vector. Since $\theta_{k}$, $B^{L}$, and $B^{W}$ are all known, $\alpha =\frac{-\theta_{k}B^{L}}{\ln2}$, and $\beta =\frac{-\theta_{k}B^{W}}{\ln2}$; the Lagrangian function in Equation (29) is expressed as follows:

(30) $$L\left(P_{k}^{L},P_{k}^{W},\lambda_{1}\right)=E_{\gamma_{k}^{}}\left\{{\left(1+P_{k}^{L}\gamma_{k}^{L}\right)}^{\alpha }{\left(1+P_{k}^{W}\gamma_{k}^{W}\right)}^{\beta }\right\}-\lambda_{1}\left(P_{k}^{L}+P_{k}^{W}-P_{k}^{\max}\right).$$

Setting the result to 0 yields the following results:

(31) $$\alpha \gamma_{k}^{L}{\left(1+P_{k}^{W}\gamma_{k}^{W}\right)}^{\beta }{\left(1+P_{k}^{L}\gamma_{k}^{L}\right)}^{\alpha -1}-\lambda_{1}=0,$$

(32) $$\beta {\left(1+P_{k}^{L}\gamma_{k}^{L}\right)}^{\alpha }{\left(1+P_{k}^{W}\gamma_{k}^{W}\right)}^{\beta -1}\gamma_{k}^{W}-\lambda_{1}=0,$$

(33) $$P_{k}^{L}+P_{k}^{W}-P_{}^{\max}=0.$$

Finally, solving Equations (31)–(33) yields the optimal uplink power allocation:

(34) $$\left\{ \begin{array}{l} P_{k}^{L*}=\frac{B^{L}\gamma_{k}^{L}\left(1+P^{\max}\gamma_{k}^{W}\right)-B^{W}\gamma_{k}^{W}}{\gamma_{k}^{L}\gamma_{k}^{W}\left(B^{L}+B^{W}\right)}\\ P_{k}^{W*}=P^{\max}-P_{k}^{L*}. \end{array}\right.,$$

Next, we study the optimal bandwidth-allocation mechanism based on the optimal power-allocation mechanism.

(2) Optimal bandwidth-allocation mechanism

After determining the optimal uplink power allocation, based on the impact of a service on the data-packet transfer rate, we reasonably allocate the bandwidth to obtain the total maximum effective network capacity. Thus, the optimal bandwidth-allocation mechanism can be obtained by solving the following convex optimization problem:

(35) $$\underset{\underset{1\leq k\leq K}{B_{k}^{L},B_{k}^{W}}}{\arg\min}\left\{E_{\gamma_{i}^{L}}\left\{\prod_{k=1}^{K}{\left(1+P_{k}^{L*}\gamma_{k}^{L}\right)}^{\frac{-\theta_{k}^{}B_{k}^{L}}{\ln2}}\right\}+E_{\gamma_{k}^{W}}\left\{\prod_{k=1}^{K}{\left(1+P_{k}^{W*}\gamma_{k}^{W}\right)}^{\frac{-\theta_{k}^{}B_{k}^{W}}{\ln2}}\right\}\right\},$$

(36) $$s.t.\;\sum_{k=1}^{k=K}B_{k}^{L}\leq B_{}^{L},$$

(37) $$\sum_{k=1}^{k=K}B_{W}^{L}\leq B_{}^{W},$$

(38) $$B_{k}^{L},B_{W}^{L}\geq 0.$$

The constraint in Equation (36) indicates that the bandwidth occupied by all the services on the NR link is smaller than the total uplink bandwidth of the NR. A similar constraint in Equation (37) indicates that the bandwidth occupied by all the services on the WLAN link is smaller than the total uplink bandwidth of the WLAN. Additionally, the constraint in Equation (38) indicates that each service has an opportunity to upload data.

To solve the convex optimization problem, the optimal bandwidth-allocation mechanism should satisfy $\sum_{k=1}^{k=K}B_{i}^{L*}=B^{L}$. The reason is as follows: with $B_{i}^{L*}$ as the optimal uplink bandwidth-allocation mechanism and $E_{L}^{*}\left(\theta_{i}\right)$ as the maximum effective network capacity of the NR, if $\sum_{k=1}^{k=K}B_{i}^{L}<B^{L}$, the surplus bandwidth remains in the network. Any additional capacity owing to the remaining network bandwidth will reduce the objective function. Thus, the optimal bandwidth-allocation mechanism should satisfy $\sum_{k=1}^{k=K}B_{i}^{L*}=B^{L}$.

Further, the sub-gradient algorithm is used to determine the optimal solution of the optimal bandwidth-allocation mechanism. The Lagrangian function in Equation (35) is expressed as follows:

(39) $$\begin{array}{c} L_{2}\left(B_{k}^{L},B_{k}^{W},p,q\right)=E_{\gamma_{k}^{L}}\left\{\prod_{i=k}^{K}{\left(1+P_{k}^{L*}\gamma_{k}^{L}\right)}^{\frac{-\theta_{k}^{}B_{k}^{L}}{\ln2}}\right\}+E_{\gamma_{k}^{W}}\left\{\prod_{i=k}^{K}{\left(1+P_{k}^{W*}\gamma_{k}^{W}\right)}^{\frac{-\theta_{k}^{}B_{k}^{W}}{\ln2}}\right\}\\ +p\left(\sum_{k=1}^{k=K}B_{i}^{L}\leq B_{}^{L}\right)+q\left(\sum_{k=1}^{k=K}B_{i}^{W}-B_{}^{W}\right), \end{array}$$

where p and q are the nonnegative Lagrangian multipliers. After obtaining $L_{2}$ for the partial derivatives, $B_{k}^{L}$ and $B_{k}^{W}$, the results are set to 0 to obtain the following:

(40) $$\left\{ \begin{array}{c} \frac{\partial L_{2}}{\partial B_{k}^{L}}=-\frac{\theta_{k}^{}}{\ln2}\prod_{i=k}^{K}{\left(1+P_{k}^{L*}\gamma_{k}^{L}\right)}^{\frac{-\theta_{k}^{}B_{k}^{L}}{\ln2}}\ln\left(1+P_{k}^{L*}\gamma_{k}^{L}\right)=0,\\ \frac{\partial L_{2}}{\partial B_{k}^{W}}=-\frac{\theta_{k}^{}}{\ln2}\prod_{i=k}^{K}{\left(1+P_{k}^{W*}\gamma_{k}^{W}\right)}^{\frac{-\theta_{k}^{}B_{k}^{W}}{\ln2}}\ln\left(1+P_{k}^{W*}\gamma_{k}^{W}\right)=0. \end{array}\right.$$

The pointwise convergence of the lower bound of the Lagrangian function $L_{2}$ yields the Lagrangian dual function as follows:

(41) $$D\left(p,q\right)=\underset{\underset{1\leq i\leq K}{\left(B_{k}^{L},B_{k}^{W}\right)}}{\inf}L_{2}$$

The sub-gradient method is employed to solve the dual problem, and the Lagrangian multipliers are solved in each iteration using the following:

(42) $$\left\{ \begin{array}{l} p\left(s+1\right)=p\left(s\right)+v\left(s\right)\frac{\partial L_{3}}{\partial p},\\ q\left(s+1\right)=q\left(s\right)+v\left(s\right)\frac{\partial L_{3}}{\partial q}, \end{array}\right.$$

where *s* represents the number of iterations, $\frac{\partial L_{2}}{\partial p}$ and $\frac{\partial L_{2}}{\partial q}$ represent gradients, and v(s) represents the gradient step size for each iteration, *s*.

In summary, Equation (20) is an optimization problem, for maximizing the total effective capacity of users in the system, where the limiting conditions are the bandwidth and the user power. As the two resources do not affect each other, the total optimization problem is decomposed into two sub-problems, as Equations (25) and (35). Equations (28) to (34) detail the process of solving the sub-problem in Equation (25), using the Lagrangian equation method. Equations (39) to (42) detail the solution methods of the sub-problem in Equation (35), using the sub-gradient method.

The procedure to solve the optimal bandwidth allocation is presented as Algorithm 1.
**Algorithm 1.** Optimal bandwidth allocation using the sub-gradient method.1. Declare, instantiate, and initialize *s* = 0 as a counter of the number of iterations s according to (35).2. Input the initial values of $B_{k}^{L}$, $B_{k}^{W}$, p, q, and $\theta_{k},1\leq k\leq K$.3. Initialize maximum tolerance threshold (ε) and v(0).4. Calculate gradients $\frac{\partial L_{2}}{\partial B_{k}^{L}}$$\;\mathrm{and}\;\frac{\partial L_{2}}{\partial B_{k}^{W}}$ according to (39) and (40).5. Solve $\frac{\partial L_{2}}{\partial B_{k}^{L}}=0$
$\mathrm{and}\;\frac{\partial L_{2}}{\partial B_{k}^{W}}=0$ then calculate current optimal values $B_{k}^{L*}\left(s-1\right)$$\;\mathrm{and}\;B_{k}^{W*}\left(s-1\right)$$\;\mathrm{based}\;\mathrm{on}\;P_{L}^{*}$$\;\mathrm{and}\;P_{W}^{*}$, as obtained from the optimal power-allocation mechanism described by (34).6. Calculate the current optimal effective network capacity, $E_{i}^{*}\left(s-1\right)$.7. Increment the counter for the next iteration of the algorithm: s=s+1.8. Continue solving $\frac{\partial L_{2}}{\partial B_{k}^{L}}=0$$\;\mathrm{and}\;\frac{\partial L_{2}}{\partial B_{k}^{W}}=0$ and obtaining current optimal values $B_{i}^{L*}\left(s\right)$$\;\mathrm{and}\;B_{i}^{W*}\left(s\right)$ according to optimal power allocations $P_{L}^{*}$$\;\mathrm{and}\;P_{W}^{*}$.9. $\mathrm{Calculate}\;E_{i}^{*}\left(s\right)$.10. Update p and q according to the iteration formula in (42).11. $\mathrm{Loop}\;\mathrm{until}\;\left|E_{}^{*}\left(s\right)-E_{}^{*}\left(s-1\right)\right|\leq \varepsilon$.12. End.

## 4. Simulation Results and Analysis

### 4.1. Simulation Parameters

In this study, the feasibility of using the proposed MSTT algorithm and the network capacity realized with it were experimentally verified through simulations. To this end, an NR network was deployed as a macro-BS, thereby representing 3GPP scenarios. Likewise, a WLAN was deployed as a micro-BS, representing non-3GPP scenarios. In the proposed simulation platform, all UEs supported ATSSS, and the signaling interaction between the 3GPP and non-3GPP networks followed ATSSS rules. In our system simulation, the network architecture, protocols, signaling, and simulation scenarios were constructed in full compliance with 3GPP standards. During the simulation, all UEs were randomly distributed, moved at a speed of 30 km/h, and changed position every 100 TTIs. The maximum transmitted power of each UE was 21 dBm. The handover time for the UE in the candidate set was called the intra-network handover delay, which was set to 10 m. If the BS to which the UE was handed over to was not in the candidate set, the handover time was called the inter-network handover delay and was set to 50 m.

Additionally, we simulated a configuration wherein no UE demonstrated multi-path operation. This configuration is referred to as single connectivity (SC). In the SC scenario, the UE *u* connected to only one path, with maximum $M_{u,c}$. According to the 3GPP and the IEEE 802.11 g standards, the proposed channel model complied with 3GPP 38.901 The cellular network structure was represented by seven NR BSs, and three WLAN APs were evenly distributed under each NR BS. The statistical window of the average load of the BS was 200 m. The under-loaded and overloaded scenarios were simulated by distributing different numbers of UEs for each BS, and an unevenly loaded scenario comprised a random combination of the under-loaded and overloaded scenarios.

Each user-service type employed an FTP model with a service data-packet arrival rate α. The total uplink bandwidths of the NR network and WLAN links were 10 M Hz and 22 M Hz, respectively. Additionally, “normalized effective network capacity” in the simulation results referred to the effective network capacity in Hz/s.

The most important parameters used in the simulations and their corresponding values are listed in Table 1.

### 4.2. Simulation Results

During the simulation, the 5% of users who demonstrated the lowest throughput were considered cell-edge users. The simulation results reveal that the average throughput realized by the SC NR cell-edge users was 0.60 Mbps, while that of the corresponding multi-path users was 9.52 Mbps. This confirms that the ATSSS feature significantly improves the throughput of cell-edge users. Next, we focused our analysis on user satisfaction. We used the bandwidth and power average distribution (AD) scheme as a comparison scheme. 3GPP R-17 TS 23.501 defines three steering modes supported by ATSSS technology. Correspondingly, there are three types of network switching. The three modes are: active-standby, smallest delay, and load-balancing [52]. Among them, the AD algorithm is one of the load-balancing steering modes. In load-balancing steering mode, each access network receives a percentage of Multi-Access PDU (MA-PDU) session data, depending on the assigned weighting factor. The AD algorithm equally distributes the power of each UE in the calculation of the weight factor.

**Figure 5:**
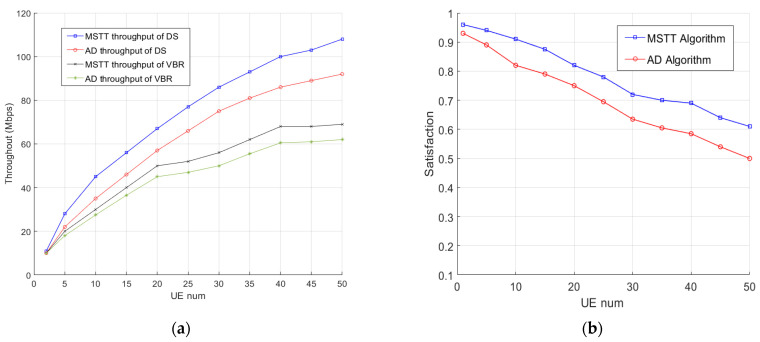
(**a**) Average throughput of VBR and DS services. (**b**) Average satisfaction of VBR and DS services.

We first analyzed the CBR service. The simulation results are shown in Table 2. For the AD scheme, the number of users meeting the CBR threshold reached 100%, which means that all CBR services were satisfied. In the MSTT scheme, this value slightly dropped, but remained above 99.64%. This small sacrifice brought about a significant increase in the average throughput of VBR and DS services, as shown in Figure 5a. Figure 5b shows the mean value of satisfaction for VBR and DS services in the system. It shows that, compared with the AD scheme, the MSTT scheme always greatly improves the satisfaction level of the UEs’ services in the system to better meet their different requirements.

**Figure 6:**
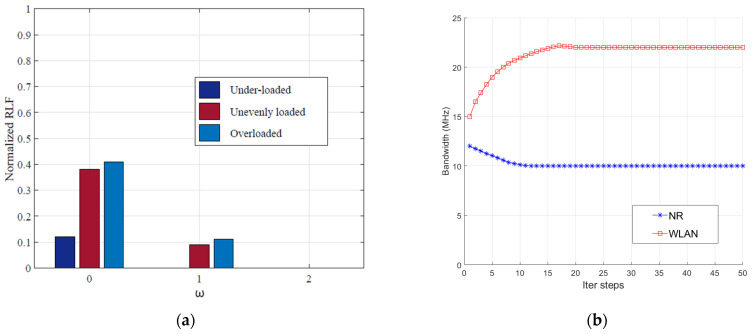
(**a**) Normalized RLF for under-loaded, unevenly loaded, and over-loaded scenarios for different ω; (**b**) Bandwidth distribution with the number of iterations.

The under-loaded and over-loaded scenarios were simulated by distributing 5 UEs and 15 UEs, respectively, for each cell, and an unevenly loaded scenario corresponded to a random combination of the under-loaded and over-loaded scenarios. RLF is one of the main sources of service disruption and is critical to future mobile networks. Figure 6a depicts the normalized RLF corresponding to the under-loaded, unevenly loaded, and over-loaded scenarios for different ω. The figure shows that, for the under-loaded scenario, the RLF is completely solved at ω=1. For the unevenly loaded and over-loaded scenarios, the RLF is completely solved at ω=2. This is because as ω increases, the condition that BS c continues to remain in the candidate set becomes relaxed, and the probability of it remaining in the candidate set of UE u increases.

As an example of convergence, we randomly selected a UE in the system and plotted the evolution of the corresponding bandwidth. Figure 6b shows that, after 10 iterative steps, the bandwidth allocation of the NR BS finally converges to 10 MHz, and after 20 iterative steps, that of the WLAN converges to 22 MHz. This indicates that the MSTT scheme is convergent through a limited number of iterations.

**Figure 7:**
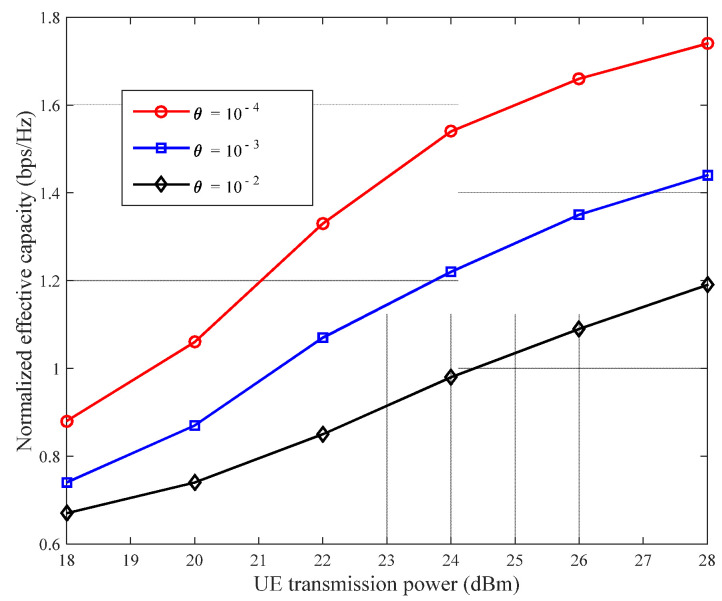
Relationship between size of DQoS index θ and normalized effective network capacity.

As already mentioned in the Introduction section, the size of the DQoS index, θ, reflects the strictness of the network requirements for QoS. Figure 7 shows the effect of different θ values on the normalized effective network capacity. Evidently, the larger the θ value, the smaller the normalized effective network capacity. This is because, for larger DQoS indices, more network resources are consumed to guarantee the service QoS index, thereby reducing the normalized effective network capacity [43]. This result is consistent with that obtained theoretically. Additionally, the total effective network capacity increases with increasing transmission power.

The network capacity obtained using the proposed MSTT algorithm was evaluated and compared with those obtained using the always-best-connected (ABC) and fixed-ratio power-allocation algorithms. The ABC algorithm is an extended steering mode that is based on the smallest delay. The purpose of the ABC algorithm is to always connect to the optimal link. If it is assumed that the link with the smallest delay is the optimal link, the ABC algorithm is equivalent to smallest delay switching. The power-allocation algorithm essentially obtains the maximum-power SC, with a core concept that includes all the BSs. Thus, the user always connects to the BS receiving the largest RSRP and uses the maximum UE transmit power when uploading data. The main advantage of this ABC power-allocation algorithm is that, although the user can obtain a higher SNR, frequent network switching increases data transmission delay and greatly deteriorates the user’s experience of a delay-sensitive service. In the fixed-proportion power-allocation algorithm, users can allocate certain proportions of power to an NR link and a WLAN link. In our simulation, F(x) was used to represent x% of the total user power allocated to the NR.

**Figure 8:**
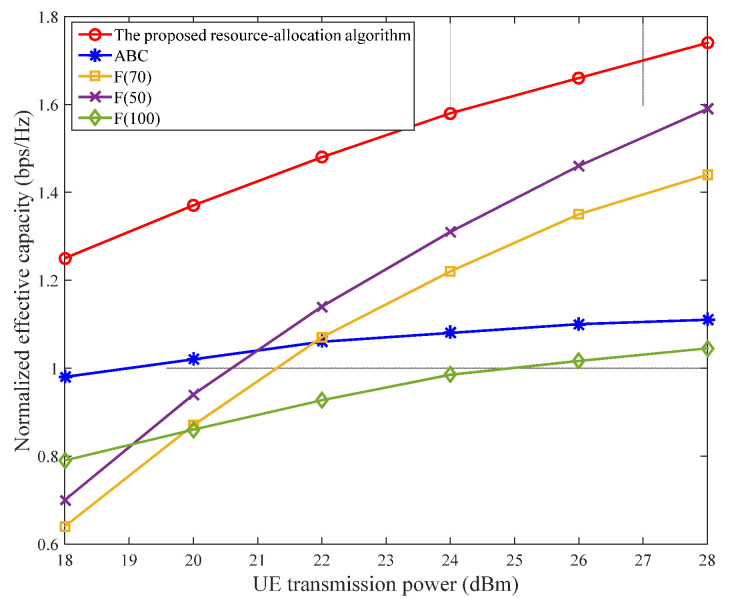
Relationship between normalized effective network capacity and UE-transmit power for evenly distributed network users.

Figure 8 shows a comparison of the network capacities obtained based on the proposed MSTT algorithm, the ABC algorithm, and the fixed-ratio power-allocation algorithm with varying user-transmit power. The network users are evenly distributed, with $\theta =10^{-3}$. F(100), F(70), and F(50) represent fixed-ratio power-allocation algorithms, which indicate that users have allocated 100%, 70%, and 50% of the total network power to the NR, respectively. F(50) also represents the AD algorithm.

Evidently, the NWA dual network connectivity is superior to a single network connection. With increasing user-transmit power, the network capacity advantages of dual connectivity became more pronounced given that dual connectivity can effectively offload data on overloaded BSs, and increase the overall effective network capacity via the second link, thereby improving the network capacity. Additionally, the simulation using F(100) resulted in the worst network capacity because the large bandwidth and high capacity of the WLAN were not fully utilized. The simulation results also indicate that the network capacity achieved using the proposed MSTT algorithm is always better than those obtained using other algorithms, because the MSTT algorithm distributes power as freely and reasonably as possible to achieve the optimal network capacity.

**Figure 9:**
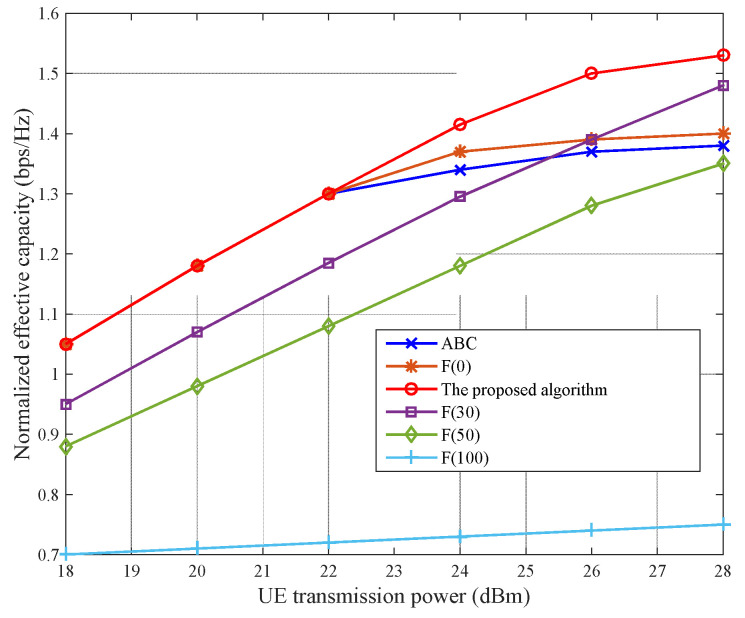
Relationship between normalized effective network capacity and UE-transmit power for unevenly distributed network users.

Unlike the even distribution of network users in the previous scenario, users were unevenly distributed close to the WLAN APs to simulate the indoor scenario. The other simulation parameters were the same as those shown in Figure 8, and the simulation results are shown in Figure 9. Evidently, the proposed MSTT algorithm always maintains the best network capacity of the three employed algorithms. Furthermore, the WLAN exhibits very similar network capacities for the ABC and F(0) power-allocation algorithms. However, the user-transmit powers are different; this finding fully reflects the high network capacity of the WLAN. Moreover, with increasing user-transmit power, some of the power allocated to the NR can increase the effective network capacity, owing to saturation of the WLAN, and sharing data on the NR may continue to increase the effective network capacity. However, the network capacity was lowest when all the power was allocated to the NR (i.e., the F(100) power-allocation algorithm) because users were too far away from the NR BS and the signal attenuation was too high. The network capacity was observed to degrade if the NR link continued to transmit data.

Taken together, the simulation results shown in Figure 8 and Figure 9 suggest that, although the use of a fixed-ratio (i.e., proportional) power-allocation algorithm can enhance the effective network capacity under certain conditions, it is difficult to define x% that is suitable for all situations; thus, network robustness cannot always be guaranteed.

**Figure 10:**
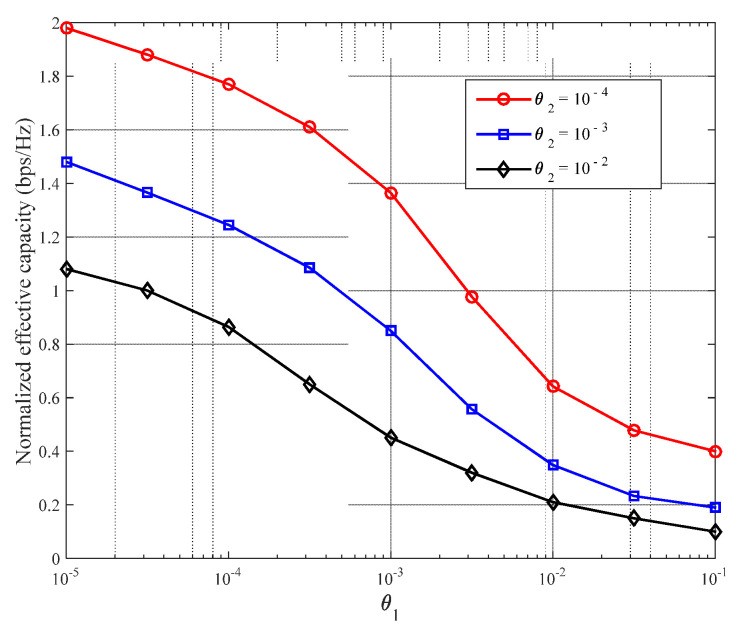
Relationship between effective network capacity and QoS index for simultaneous multiple services.

Further, the relationship between the effective network capacity and a service, when one of the service QoS indices is fixed, is shown in Figure 10. The figure shows that the normalized effective network capacity decreases as the DQoS index of another service increases when a particular service QoS index is fixed. Furthermore, when two service QoS indices simultaneously increase, the total effective network capacity decreases rapidly.

## 5. Conclusions

We implemented and optimized the traffic scheduling in an NWA network to improve user satisfaction and comply with service DQoS. Unlike the throughput in an actual wireless channel, based on multiservice provision, network throughput was evaluated using the effective network capacity obtained under DQoS constraints. First, the smart terminal selected the data transmission path according to the service type. Then, the traffic scheduling optimization problem was transformed into a constrained convex optimization problem, which was, in turn. subdivided into power- and bandwidth-allocation optimizations. The user satisfaction and network capacity achieved using the proposed MSTT algorithm were evaluated through simulation experiments which showed an improvement in user satisfaction. Additionally, the network capacity obtained using the proposed MSTT algorithm was always superior to those achieved using the ABC and fixed-ratio power-allocation algorithms. This is because the proposed algorithm distributes power as freely and reasonably as possible to achieve the optimal network capacity. Furthermore, although the use of a fixed-ratio (i.e., proportional) power-allocation algorithm can enhance the effective network capacity under certain conditions, it is difficult to define percentage of the total user power allocated to the NR link suitable for all situations; thus, network robustness cannot always be guaranteed. This is a topic for further research. The proposed traffic scheduling algorithm will be beneficial for optimizing 5G network resources, such as network bandwidth and power.

## Figures and Tables

**Table 1:** Simulation parameters and corresponding values.

Parameter	Value	
Carrier frequency	NR WLAN	3.5 GHz 5 GHz
Propagation path-loss model	NR WLAN	Urban macro cellular (Uma)Indoor
Bandwidth	NR WLAN	10 MHz 22 MHz
NR inter-site distance (ISD)	40 m	
WLAN AP in a cell	3	
Maximum transmit power (UE)	21 dBm	
UE moving speed	30 km/h	
Inter-network handover delay	50 ms	
Intra-network handover delay	10 ms	
File-transfer protocol (FTP) service data-packet arrival rate α	1	
Packet size	4 M	
Window size (mathematically obtained from expectation statistics)	200 ms	
$Td_{cbr}$	8 kbps	
$Td_{ds}$	20 Mbps	
$Td_{vbr}$	12 Mbps	
ε	0.001	
s	1	

**Table 2:** Satisfaction of CBR.

UE Num	10	20	30	40	50
D utility (%)	100	100	100	100	100
MDT uility (%)	99.658	99.765	99.639	99.772	98.683
